# Pregnancy-Associated Cardiomyopathy and Long-Term Cardiac Phenotype in Women With Truncating Titin Variants

**DOI:** 10.1016/j.jacadv.2026.102857

**Published:** 2026-06-24

**Authors:** Anna Isotta Castrini, Nina Eide Hasselberg, Eivind Westrum Aabel, Cecilie Bugge, Christian Kullmann Five, Knut Erik Berge, Mette Elise Estensen, Kristina Hermann Haugaa

**Affiliations:** a ProCardio Center for Innovation, Department of Cardiology, Oslo University Hospital, Rikshospitalet, Oslo, Norway; b Division of Medicine, Department of Cardiology, Hospital in Bærum, Vestre Viken, Gjettum, Norway; c University of Oslo, Oslo, Norway; d Unit for Cardiac and Cardiovascular Genetics, Department of Medical Genetics, Oslo University Hospital, Oslo, Norway

**Keywords:** dilated cardiomyopathy, peripartum-cardiomyopathy, pregnancy, titin

## Abstract

**Background:**

Peripartum cardiomyopathy (PPCM) occurs in 0.02% to 0.1% of live births in European countries. Truncating variants in the Titin gene (TTNtv) are the most common genetic findings in women with PPCM. However, data on pregnancy-associated cardiomyopathy (PAC) and pregnancy tolerance among TTNtv-positive women are limited.

**Objectives:**

The objectives of the study were to report maternal cardiac outcomes during pregnancy and postpartum and fetal outcomes in women with TTNtv and to explore the effect of parity on long-term cardiac phenotype.

**Methods:**

We conducted an ambispective cohort study including consecutive TTNtv genotype-positive women, regardless of phenotype, with ≥1 cardiological evaluation. Data on pregnancies and maternal and fetal outcomes were collected from questionnaires and medical records. Arrhythmic burden and device implantation were recorded. Left ventricular dimensions and function were assessed by echocardiography.

**Results:**

A total of 107 TTNtv-positive women were included (age 43 [IQR: 32-55] years, 17% probands). Among 78 women with prior pregnancies, 206 pregnancies resulted in 167 (81%) live births. PAC occurred in 3 of 167 live births and in 3 of 76 women, corresponding to a cumulative incidence of 1.8% and 3.9%. All affected women recovered completely with medical therapy. Remaining pregnancies were largely uncomplicated, without increased maternal or fetal adverse events. At median of 18 years (IQR: 9-31) after pregnancy, no differences were observed between parous and nulliparous women in arrhythmic burden, cardiac function, or ventricular dimensions.

**Conclusions:**

In this TTNtv-positive cohort, PAC incidence appeared higher than reported PPCM rates in the general European population. However, pregnancy was well tolerated and was not associated with adverse long-term cardiac remodeling.

Peripartum cardiomyopathy (PPCM) is a life-threatening heart disease, characterized by heart failure with left ventricular ejection fraction (LVEF) below 45% occurring in late pregnancy or during the first months after delivery, abortion, or miscarriage, with no other explainable cause.^1^ PPCM is a major cause of maternal death responsible for 6% to 11% of cardiac transplantations among women in the United States.^2^ Pre-eclampsia, twin gestation and advanced maternal age are the strongest known risk factors.^1^^,^^3^ The incidence of PPCM differs widely according to geographic area and ethnic background. In European countries, the incidence of PPCM was reported to be 0.02% to 0.1% of live births.^4^

One of the key hypotheses on pathogenesis of PPCM includes cardiac angiogenic imbalance caused by higher oxidative stress and antiangiogenic signals; however, the role of multiple contributing factors driving disease progression remains unclear.^5^ PPCM and dilated cardiomyopathy (DCM) share some clinical features, including symptoms, impaired cardiac systolic function, enlarged cardiac dimensions, and nonspecific histologic findings on endomyocardial biopsy.^3^ Variants that shorten, or truncate, the Titin protein (TTNtv) are the most common genetic cause of DCM, found in up to 15% to 25% of familial DCM cases.^6^ TTNtv variants were found to be common in families with both PPCM and DCM.^7^ The hypothesis of a genetic predisposition in PPCM was previously tested and TTNtv variants were described as the most prevalent genetic variants in PPCM.^3^^,^^8^ On the other hand, the incidence of heart failure with left ventricular systolic dysfunction and pregnancy tolerance during pregnancy/postpartum in women carrying a TTNtv is unknown. In the present study, we referred to heart failure with left ventricular systolic dysfunction occurring during pregnancy/peripartum in women with TTNtv as pregnancy-associated cardiomyopathy (PAC). Although maybe related, PAC is a condition distinct from PPCM which requires exclusion of an identifiable cause of cardiomyopathy, including genetic predisposition.^1^

We aimed to report the incidence of PAC, maternal cardiac outcomes associated with pregnancy and postpartum period, and fetal outcomes in TTNtv genotype positive women, including both phenotype positive and negative individuals. Furthermore, we aimed to explore the effect of parity on long-term cardiac phenotype in this population.

## Methods

### Study population

We performed an ambispective cohort study, including consecutive women heterozygous for a pathogenic or likely pathogenic TTNtv. All patients were referred to our outpatient clinic for genetic cardiac diseases at Oslo University Hospital, Rikshospitalet after TTNtv genetic diagnosis, and all underwent at least 1 cardiological evaluation. Race and ethnicity were not systematically collected. Given the single-center design in Norway, the study population was likely predominantly of European ancestry. Probands were defined as the first individuals in a family, without known history of TTNtv-related disease, who sought medical attention due to clinical manifestation of TTNtv-related cardiomyopathy and underwent genetic testing. Both probands and family members diagnosed with a TTNtv through family cascade screening were included in our study, independently of the presence of a cardiac phenotype. Frequency of clinical visits and further follow-up were scheduled on clinical indication. The last follow-up was defined as the last cardiological consultation including an echocardiography examination and electrocardiogram (ECG) at our center, before August 2024. The postpartum period was defined as the first 6 months after delivery.

All patients gave written informed consent. The study was conducted in accordance with the Declaration of Helsinki and was approved by the Regional Committees for Medical Research Ethics.

### Genetic analysis

Before 2018, probands with suspected DCM were tested by Sanger sequencing for selected genes, including known TTNtv. Family members were investigated with cascade genetic screening.^9^ From 2018, probands with DCM were tested by next-generation sequencing (TruSight Cardio Sequencing Kit, Illumina Inc).^10^ The pathogenicity of the genetic variants was evaluated according to the guidelines of the American College of Medical Genetics and Genomics and the Association for Molecular Pathology.^11^ Patients heterozygous for pathogenic or likely pathogenic variants were considered genotype positive.

### Pregnancy and postpartum data

TTNtv-positive women with and without a history of pregnancy were included. Between August 2022 and August 2024, a health questionnaire on pregnancy and the postpartum period was administered to the patients at clinical visit, by mail, or via structured telephone interviews. Data collected through the questionnaire were retrospective and self-reported. The questionnaire recorded data on pregnancies, the number of miscarriages (before and after 12 weeks of gestation), and parity (Figure 1). In addition, obstetrical complications, together with symptoms and medical therapy during pregnancy and the postpartum period, were collected. Fetal outcomes, history of hospitalizations, and visits to emergency rooms or outpatient clinics during pregnancy and the postpartum period were self-reported through the questionnaire. Awareness of the TTNtv at time of the pregnancy was recorded. For patients followed at our center during pregnancy, data on maternal and fetal outcomes were collected retrospectively from available patients’ medical records.

**Figure 1:**
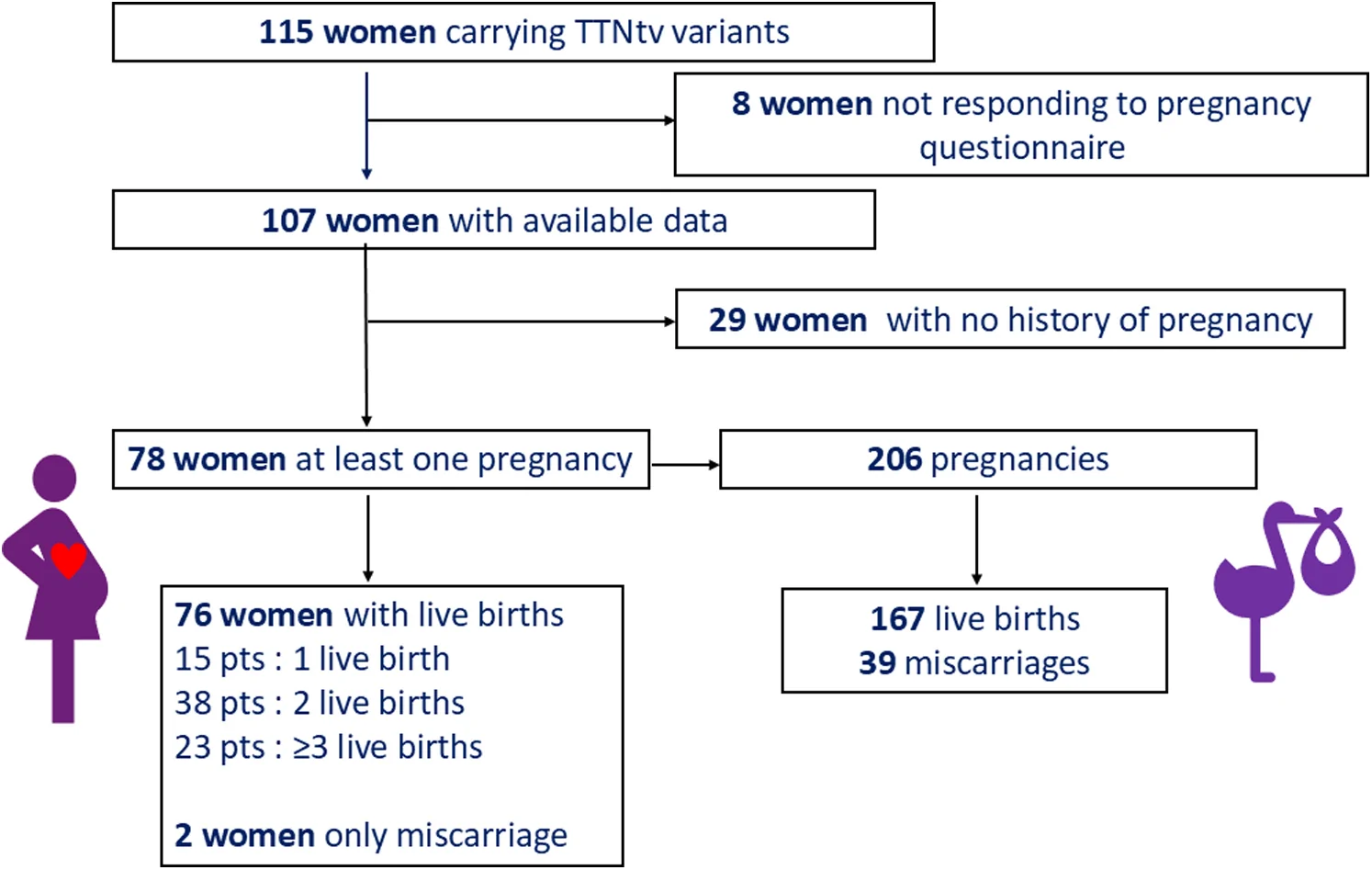
Study Cohort and Number of Pregnancies in Women With Titin Truncating Variants Flow diagram illustrating the identification of women carrying TTN truncating variants and the derivation of the final pregnancy cohort. Among 115 women with TTNtv, 8 did not respond to the pregnancy questionnaire, leaving 107 women with available data. Of these, 29 had no history of pregnancy. The remaining 78 women experienced at least 1 pregnancy, accounting for 206 pregnancies in total. These pregnancies resulted in 167 live births and 39 miscarriages. Among women with prior pregnancies, 76 had at least 1 live birth (15 women had 1 live birth, 38 had 2 live births, and 23 had ≥3 live births), whereas 2 women experienced miscarriages only. TTNtv = titin truncating variant.

PAC was defined as new-onset of symptoms and signs of heart failure with LVEF <45% occurring during pregnancy or within 6 months postpartum in women with an identifiable disposition for cardiomyopathy. Hence, clinically and structurally PAC is in line with the classical definition of PPCM,^1^ but with an identified underlying genetic disposition.

For patients who developed PAC, all available information on the clinical history and outcome after heart failure episodes were reported.

### Arrhythmias and echocardiographic examinations

The cardiological consultation included medical history, 12-lead ECG, and echocardiographic examination. A subgroup of patients underwent 24-hours Holter ECG recording based on clinical indication. Data on atrial fibrillation (AF), nonsustained (NSVT)/sustained ventricular tachycardia (VT) and implantable cardioverter-defibrillator/cardiac resynchronization therapy defibrillator implantation were recorded. AF was defined according to the latest guidelines.^12^ Ventricular tachycardias with a rate ≥110/min were defined as NSVT or sustained VT if lasting <30 or ≥30 seconds, respectively. Data on left ventricular end-diastolic diameter (LVEDD) and LVEF by echocardiography were measured according to guidelines.^13^ These clinical and imaging data were predominantly recorded retrospectively from medical records, whereas, in a subset of patients, data were prospectively collected during follow-up. Left ventricular systolic dysfunction during pregnancy and postpartum was defined as LVEF <45%, consistent with the definition of PPCM. For long-term follow-up after pregnancy, left ventricular systolic dysfunction was defined as LVEF <50% according to current heart failure guidelines.^14^ Outcomes of heart transplantations and death from all causes were reported.

### Outcomes

The primary outcome was the incidence of PAC among pregnancies resulted in live birth in TTNtv positive women. Secondary outcomes included maternal cardiac symptoms during pregnancy and postpartum (dyspnea, syncope, and palpitations), maternal cardiac complications (including maternal death), obstetric outcomes (pre-eclampsia, gestational diabetes, and mode of delivery), and fetal outcomes (fetal death, low birth weight, and preterm birth).

Long-term cardiac outcomes in TTNtv carriers were evaluated according to parity, comparing parous, and nulliparous women. These long-term outcomes included arrhythmic burden and measures of left ventricular structure and function at first cardiological consultation.

### Statistical analysis

Data were presented as frequencies with percentages, mean ± SD and median [IQR] as appropriate. *P* values were calculated using the analysis of variance F-test, Fisher exact test, and Kruskal-Wallis test. Pregnancies that ended in live birth were treated as the exposure variable when evaluating the effect of pregnancy on development of the TTNtv-related cardiac phenotype. Pregnancies were analyzed as independent observations. The incidence of PAC was reported as a cumulative incidence proportion and calculated per live birth and per parous woman. Multivariable analyses were conducted using logistic regression for binary outcomes and linear regression for continuous outcomes to assess independent associations between covariates and the outcomes of interest. Covariates included in the multivariable models were selected based on clinical relevance, including age, given the age-dependent penetrance of TTNtv-related cardiomyopathy, and medical therapy as a potential modifier of left ventricular function. We performed statistical analyses using Stata SE 18.0 (StataCorp LLC). *P* values were 2-sided, and values <0.05 were considered significant.

### Data availability

The authors do not have the authority to share the data reported in the present article, due to the sensitive nature of the data collected for this study. Approval of the Regional Committee for Medical Research Ethics limits sharing data with researchers inside or outside Norway for purposes of reproducing the results or replicating the procedures. The data can be made available to additional research after formal application to the Regional Committee for Medical Research Ethics and explicit consent obtained from every study participant.

## Results

### Study population

Participants were referred to their first cardiological consultation after TTNtv genetic diagnosis between November 2001 and August 2024. We included 115 TTNtv-positive women (Figure 1). In total, 107 (93%) completed the health questionnaire on pregnancy and the postpartum period (Figure 1). The median age at first cardiological consultation was 40 (IQR: 30-54) years and follow-up time was a median of 1 year (IQR: 1-3; range 1-23) from the first to last cardiological consultation at our center. Specific TTNtv variants are reported in Supplemental Table 1. At the last follow-up, 78 (73%) TTNtv-positive women had been pregnant at least once, whereas 29 (27%) women had never experienced pregnancy or miscarriage (Figure 1). Among the 78 women with at least 1 pregnancy, 76 experienced live births, whereas 2 experienced miscarriage only. The total number of pregnancies was 206, of which 167 (81%) ended in live birth and 39 (19%) ended in miscarriage (Table 1, Figure 1).

**Table 1:** Maternal and Fetal Outcomes in 206 Pregnancies in 107 Women With TTNtv

Number of pregnancies per woman	2 [0-3]
Age at first live birth	27 [23-31]
Age at PPCM	29 [21-30]
Miscarriage <12 wk	30 (15)
Miscarriage >12 wk	9 (5)
Maternal cardiovascular symptoms and complications during pregnancy/peripartum in pregnancies ended with a live birth, N = 167	
Maternal mortality	0 (0)
PAC	3 (2)
Pre-eclampsia	2 (1)
Gestational diabetes	1 (1)
Syncope	3 (3)
Palpitations	19 (12)
Dyspnea	5 (3)
Adverse obstetric outcomes during delivery	
Caesarean section∗	14 (9)
Induced vaginal delivery for cardiac reason	1 (1)
Emergency CS for cardiac reason	1 (1)
Adverse fetal outcomes	
Fetal or neonatal death <1 wk	1 (1)
Low birth weight (<2,500 g)	4 (3)
Preterm birth (<37 weeks)	2 (1)

Values are median [IQR] or n (%).

PAC = pregnancy-associated cardiomyopathy; PPCM = peripartum cardiomyopathy; TTNtv = truncating variant of Titin.

∗ Caesarian section (CS) experienced at least ones (14 women deliver at least 1 time with caesarian section).

In the 76 parous women, the median number of live births was 2 (range 1-4), with an age at first delivery of 27 (IQR: 23-31) years. The majority of patients (n = 71, 93%) experienced pregnancy before the genetic diagnosis of TTNtv, a median of 18 years (IQR: 9-31) earlier. Six women were diagnosed with a TTNtv during pregnancy or the postpartum period, of whom 2 (3%) underwent genetic testing due to a diagnosis of PAC.

### Maternal and fetal outcomes during pregnancy and postpartum

Among the 76 parous women, 27 (36%) reported symptoms during pregnancy including palpitations, syncope, and dyspnea (Table 1). Three (4%) patients were referred to a cardiologist and hospitalized during pregnancy due to cardiac symptoms and were diagnosed with PAC. They all started heart failure medical treatment. During pregnancy and/or the postpartum period, 5 (7%) patients were treated with beta-blockers, mostly due to palpitations. None were diagnosed with AF, NSVT, or VT during pregnancy. No patient died or required heart transplantation during pregnancy or postpartum.

One (1%) patient had a twin pregnancy and 2 (3%) experienced pre-eclampsia, none of whom developed PAC. A total of 147 (88%) of deliveries were vaginal and uncomplicated (Table 1).

Fetal or neonatal adverse outcomes were reported in 5 (3%) live births (Table 1). These included 1 (1%) fetal/neonatal death, 2 (3%) low birth weight (<2,500 g), and 4 (1%) preterm births (<37 weeks).

### Incidence and outcome of PAC in TTNtv patients

Among the 167 pregnancies ending in live birth, 3 pregnancies in 3 unique patients were complicated by PAC, giving a cumulative incidence of PAC of 1.8% (95% CI: 0.6% to 5.1%) of total live births and of 3.9% (95% CI: 0.8% to 10.8%) among parous TTNtv women. The median age at PAC diagnosis was 29 (IQR: 21-30) years. Timing of symptom onset occurred in late pregnancy or early postpartum in all cases. All PAC cases were singletons, and none was complicated by pre-eclampsia. All 3 patients were probands in their family and none were aware of TTNtv genetic diagnosis at time of pregnancy.

*Patient #1* was 29 years old, primipara in her first singleton pregnancy. No previous relevant cardiac history or family history of cardiac diseases was reported. The pregnancy was reported asymptomatic and ended with uncomplicated vaginal delivery. She developed dyspnea with orthopnea 12 days postpartum. The echocardiographic examination revealed left ventricular dilatation (LVEDD 71 mm) with systolic dysfunction (LVEF 37%) and the patient was diagnosed with PPCM. No genotyping was performed at time of PPCM diagnosis (2001). Therapy with beta-blocker and angiotensin-converting enzyme (ACE) inhibitor was started, together with anticoagulation to prevent thromboembolism. Systolic function normalized a few months after initiation of medical therapy. No adverse fetal outcomes were reported. Nineteen years later, the patient’s brother was diagnosed with DCM and TTNtv variant, and our patient underwent family genetic screening with a positive result. We therefore reclassified the clinical diagnosis as PAC after the patients tested positive for a TTNtv variant. At last follow-up, 23 years after PPCM diagnosis, our patient was asymptomatic with normalized cardiac dimensions and function. She had no subsequent pregnancies.

*Patient #2*, primipara, was 21 years old at time of her first singleton pregnancy. She had no history of cardiac symptoms, nor relevant family history. She developed fatigue, dyspnea, and tachycardia with lower-limb edema at gestational week 32 and was hospitalized. The echocardiographic examination revealed left ventricular dilatation (LVEDD 61 mm), reduced systolic function (LVEF 40%), and elevated N-terminal pro–B-type natriuretic peptide. Genetic testing performed during the hospitalization revealed a TTNtv variant. After multidisciplinary evaluation, vaginal delivery was induced with cardiac monitoring. She developed symptomatic sinus tachycardia during delivery and vacuum extraction was applied in the expulsion phase. No supraventricular or ventricular arrhythmias were recorded at 24-hour ECG monitoring. After delivery, therapy with beta-blocker and ACE inhibitor was started. No adverse fetal outcome was reported. LVEF remained subnormal during follow-up and was 46% 1 year after PPCM. She had no subsequent pregnancies.

*Patient #3* was 30 years old at time of a singleton pregnancy. She had previously experienced 2 early miscarriages (before gestational week 12). She was under regular follow-up for a mild ventricular septal defect, without signs of hemodynamic effects. She delivered by cesarean section due to obstetric indication 1 day after the estimated due date. No adverse fetal outcome was reported. Four days after delivery, the patient developed dyspnea and peripheral edema, with desaturation and sinus tachycardia. She was hospitalized. The echocardiographic examination revealed dilated left ventricle (LVEDD 63 mm), with severely reduced systolic function (LVEF 20%% to 25%). No changes in the hemodynamic significance of the ventricular septal defect were observed. Genetic testing performed during the hospitalization detected a TTNtv. Therapy with ACE inhibitor, beta-blocker, and diuretic agents was introduced, with good symptomatic effect. However, no significant improvement in LVEF was observed 3 months later. Therefore, breastfeeding was interrupted to enable introduction of all the conventional heart failure medications with normalization of LVEF after 6 months. She had no subsequent pregnancies.

### Long-term cardiac phenotype by parity in TTNtv-positive women

At last clinical follow-up, TTNtv-positive women were grouped according to parity (1, 2, and ≥3 live births) and compared with nulliparous women (Table 2). The median number of cardiological follow-up visits was 1 (1-3). Echocardiographic data were available in all participants, whereas arrhythmic outcomes data were missing in 9 patients. Among parous women, the last follow-up occurred at a median of 18 years (IQR: 9-31) after the first live birth. In the overall cohort, the median age at last follow-up was 44 years (IQR: 34-55), and women with higher parity were older, as expected (Table 2). No differences in medical therapy were observed between the parity groups.

**Table 2:** Clinical Characteristics, Therapy, and Echocardiographic Measurements at Last Follow-Up of 107 TTNtv-Positive Women Grouped by Parity

	Number of Live Births						
	0 (n = 31)	1 (n = 15)	2 (n = 38)	≥3 (n = 23)	*P* Value	≥1 (n = 76)	*P* Value 0 vs ≥1 Live Birth
Clinical characteristics							
Age a first live birth	NA	30 [21-35]	27 [24-31]	25 [23-27]	0.11	NA	NA
Age at last clinical follow-up	30 [24-36]	44 [30-57]	46 [38-61]	53 [46-64]	<0.01	47 [38-60]	<0.01
Probands	7 (23)	2 (13)	4 (11)	5 (22)	0.49	11 (15)	0.58
Atrial fibrillation	2 (6)	0 (0)	3 (8)	0 (0)	0.53	3 (4)	0.62
NSVT	3 (10)	1 (7)	12 (32)	1 (4)	0.02	14 (18)	0.38
VT	1 (3)	1 (7)	0 (0)	1 (4)	0.37	2 (3)	0.65
Medication and device therapy							
Sacubitril/valsartan	0 (0)	1 (7)	3 (8)	3 (13)	0.22	7 (9)	0.09
Beta-blockers	8 (25)	3 (20)	11 (29)	7 (30)	0.92	21 (28)	1.00
ACEI/ARBs	8 (26)	5 (33)	12 (32)	6 (26)	0.91	23 (31)	0.82
MRAs/SGLT2-inibithors	2 (7)	1 (7)	5 (13)	4 (17)	0.62	10 (13)	0.50
ICD/CRT-D	1 (3)	1 (7)	2 (5)	2 (9)	0.63	5 (7)	0.30
Echocardiographic examination							
LVEF (%)	53 ± 5	53 ± 4	53 ± 6	51 ± 9	0.69	53 ± 6	0.63
LVEDD (mm)	51 ± 5	51 ± 4	50 ± 6	52 ± 6	0.85	51 ± 5	0.84
EF <50%	4 (13)	3 (20)	6 (16)	5 (22)	0.82	14 (18)	0.58

Values are median [IQR], n (%), or mean ± SD. Patients with 0 live birth have never experienced pregnancy or experienced only miscarriages. Echocardiographic data were available for all participants (100%), whereas arrhythmic outcome data were missing in 9 patients. *P* value from ANOVA F-test, Fisher exact test, and Kruskal-Wallis test as appropriate.

ACEi = angiotensin-converting enzyme inhibitors; ARBs = angiotensin receptor blockers; CRT-D = cardiac resynchronization therapy defibrillator; EF = ejection fraction; ICD = implantable cardioverter-defibrillator; LVEDD = left ventricular end-diastolic diameter; LVEF = left ventricular ejection fraction; NA = not applicable; NSVT = nonsustained ventricular tachycardia; SGLT2 = sodium-glucose cotransporter 2; VT = ventricular tachycardia; other abbreviation as in Table 1.

At last follow-up, 3% of patients had experienced VT and 16% had experienced NSVT; however, none of these arrhythmias occurred during pregnancy. Women with 2 previous live births had a significantly higher prevalence of NSVT (*P* = 0.02) (Table 2). However, parity showed no significant effect on NSVT in the age-adjusted analyses (OR: 0.82; 95% CI: 0.45-1.50; *P* = 0.52), nor when comparing parous and nulliparous (*P* = 0.38) (Table 2). There were no differences in the prevalence of AF or VT, nor in implantable cardioverter defibrillators, either between parous and nulliparous women, nor across parity groups (Table 2).

At the last follow-up, the total cohort of 107 TTNtv-positive women had a mean LVEF 53% ± 6% and LVEDD 51 ± 5 mm. No significant differences in LVEDD or LVEF were observed in parous compared with nulliparous women, nor across parity groups (Table 2). In multivariable linear regression analysis, increasing age was independently associated with lower LVEF (β-0.14 per year; 95% CI: −0.23 to −0.05; *P* = 0.003), whereas parity was not associated with LVEF (β 0.66 per additional live birth; 95% CI: -0.61-1.93; *P* = 0.30). Parity was not associated with a significant change in LVEDD (β −0.14 per additional live birth; 95% CI: −1.37 to 1.08; *P* = 0.82). Results were unchanged after adjustment for medical therapy (LVEF: β 0.42 per additional live birth; 95% CI: −0.71 to 1.55; *P* = 0.46; LVEDD: β 0.01 per additional live birth; 95% CI: −1.18 to 1.19; *P* = 0.99).

Eighteen (17%) patients developed left ventricular systolic dysfunction during follow-up (LVEF 43% ± 7% and LVEDD 54 ± 6 mm). No significant difference in parity was observed between patients who developed left ventricular systolic dysfunction compared to those who did not (*P* = 0.48). The prevalence of systolic dysfunction at last follow-up did not differ between the parity groups (Table 2). Increasing parity was not significantly associated to systolic dysfunction (OR: 0.90; 95% CI: 0.53-1.64, *P* = 0.82) when adjusted for age. During follow-up, no death or heart transplantation was reported.

## Discussion

Our study showed a cumulative incidence of PAC of 1.8% among live births, and of 3.9% among parous in TTNtv-positive women (Central illustration). These numbers are considerably higher compared to the incidence of PPCM reported in the general European population, ranging from 0.02% to 0.1% of live births,^1^^,^^2^^,^^4^ underscoring the need for systematic pre-pregnancy counseling and follow-up in TTNtv-positive women.^15^ Reassuringly, the vast majority of TTNtv-positive women had uncomplicated pregnancies, deliveries, and postpartum periods, with no increase in fetal complications which may help counseling TTNtv-positive women with child wish. The rates of miscarriages and pre-eclampsia were comparable to the general European population.^16^, ^17^, ^18^ A history of previous pregnancy did not seem to affect long-term cardiac outcome.

**Central Illustration:**
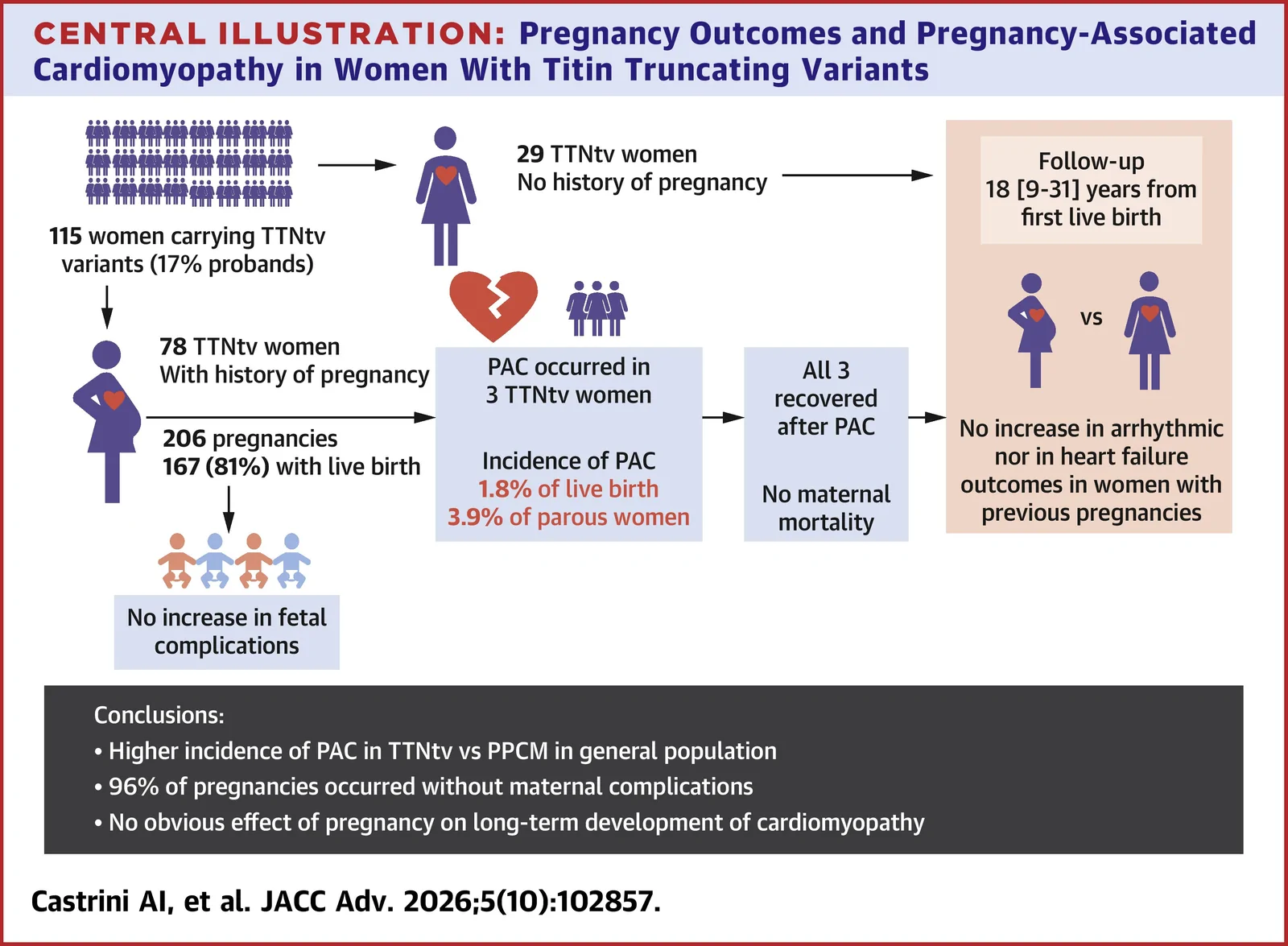
Pregnancy Outcomes and Pregnancy-Associated Cardiomyopathy in Women With Titin Truncating Variants This central illustration summarizes pregnancy outcomes and long-term follow-up in women carrying truncating variants in the Titin gene (TTNtv). Among 115 TTNtv carriers, 78 had a history of pregnancy, accounting for 206 pregnancies and 167 live births. Pregnancy-associated cardiomyopathy (PAC) occurred in 3 women, corresponding to an incidence of 1.8% per live birth and 3.9% among parous women. All affected women recovered cardiac function, and no maternal deaths occurred. Most pregnancies (96%) were not complicated by maternal cardiovascular events, and no excess fetal complications were observed. During long-term follow-up after first birth (median 18 years), parous women did not demonstrate a higher burden of arrhythmic events or heart failure compared with nulliparous TTNtv carriers. The incidence of PAC appeared higher than reported rates of peripartum cardiomyopathy (PPCM) in the general population. Overall, pregnancy was well tolerated and did not appear to accelerate long-term cardiomyopathy progression.

### PAC incidence and risk factors

This is the first study exploring the cumulative incidence of PAC among parous TTNtv-positive women and reporting fetal and maternal short- and long-term cardiac outcomes in this population. We found an incidence of PAC of 1.8% per live birth and of 3.9% per parous woman among TTNtv-positive women. A recent epidemiological study reported an incidence of PPCM of 0.0017% in the general population in Sweden.^4^ Our findings suggest that women carrying a TTNtv may have a higher risk of developing pregnancy-related left ventricular disfunction compared with the risk of developing PPCM for the general population. This represents a contextual comparison, as PPCM excludes genetic predisposition. In the pregenetic testing era, the three PAC cases identified in our cohort would likely have been classified as PPCM as no identified cause of cardiomyopathy would have been found. Previous studies suggested that DCM-related genetic variants, particularly TTNtv, may play a role in the pathogenesis of PPCM.^3^^,^^8^ The results of our study support the concept that PPCM and PAC occurring in women with a genetic predisposition may represent to distinct entities. According to the current PPCM definition, the diagnosis of PPCM should be excluded in the presence of other identifiable causes of cardiomyopathy, including a genetic predisposition.^1^ However, the genetic background of patients with PPCM is not consistently reported in clinical studies,^4^ and access to genetic testing may vary between health care systems. Therefore, it is possible that previous epidemiological studies on PPCM may report higher incidences of PPCM by including cases of PAC in patients with unrecognized underlying genetic predisposition. These considerations may support the need to further refine the current PPCM definition by explicitly addressing cases with genetic predisposition to cardiomyopathy.

One of the main pathogenetic hypothesis in PPCM includes cardiac angiogenic imbalance caused by higher oxidative stress and antiangiogenic signals in a susceptible host with inadequate protective proangiogenic defense.^5^ A genetic variant in a key sarcomere protein such as TTNtv may increase myocytes’ susceptibility to pregnancy-related stressors, like increased blood volume, cardiac output, and oxidative stress, and may therefore increase the risk of developing left ventricular dysfunction.^7^ Interestingly, the TTNtv-positive women who developed PAC in our study showed none of the other classical risk factors associated with PPCM.^1^ They were likely of European ancestry, younger than 35 years old, carrying a singleton pregnancy and without pre-eclampsia. These observations further support the hypothesis that these PAC occurring in TTNtv carriers—cases that without genetic testing might have been labeled as PPCM—may instead represent an early manifestation of genetically mediated cardiomyopathy triggered by pregnancy. Notably, none of the 3 women with PAC had family history of DCM at time of pregnancy and they developed PAC at a median age of 29 (IQR: 21-30) years. Presentation of DCM phenotype in TTNtv carriers has previously been reported at age 46 ± 13 years,^19^ suggesting that PAC may represent an early manifestation of DCM in TTNtv-positive women triggered by pregnancy.

One year after PAC, systolic function was normalized or remained mildly reduced, with no recurrent clinical heart failure or hospitalization. This is in line with results of previous reports showing that the female sex is positively associated with reverse remodeling following medical treatment in TTNtv.^19^

In 2 of our 3 patients who developed PAC, the delivery was induced to avoid maternal and obstetric complications. Therefore, we cannot exclude a higher number of complications in case of spontaneous delivery. This highlights the role of the pregnancy heart team in individualizing management of TTNtv-positive women during pregnancy.^15^

### Pregnancy and the postpartum period in the total cohort of TTNtv-positive women

Almost one-third of patients developed symptoms during pregnancy. However, the vast majority (96%) of TTNtv-positive women in our cohort had largely uncomplicated pregnancies and deliveries. Palpitations were the most common symptom reported during pregnancy, similar to reports from the general population.^20^ Except for the 3 patients with PAC, none required cardiological evaluation or hospitalization during pregnancy or in the postpartum period. The relatively low incidence of cardiac complications observed in our cohort appears consistent with the risk predicted by established pregnancy risk models such as CARPREG II^21^ and the modified WHO 2.0 (mWHO) classification.^15^ These models primarily rely on clinical parameters, including ventricular dysfunction, prior cardiac events, and functional status. None of the women in our cohort had a diagnosis of DCM or a history of cardiac events at the time of pregnancy, which may explain the relatively favorable maternal outcomes. Based on mWHO 2.0 and CARPREG II risk models, women in our cohort would be classified as mWHO class I with a CARPREG II score of 0, categories associated with no detectable increase in maternal mortality risk and no or only mild increase in maternal morbidity.^15^^,^^21^ Importantly, none of the women in our cohort had a diagnosis of DCM before pregnancy; therefore, our results cannot be used to inform risk stratification in TTNtv-positive women with overt DCM.

Interestingly, the incidence of pre-eclampsia in our cohort was low (3%). Notably, none of the women who developed pre-eclampsia experienced PAC, despite the well-established association between pre-eclampsia and an increased risk of future heart failure.^22^ This might support the hypothesis that PAC and PPCM represent distinct clinical entities.

The hemodynamic changes related to pregnancy and peripartum seemed to be well tolerated by most of TTNtv-positive women. This is in line with the results of previous studies on pregnancy tolerance in women with genetic variants predisposing to cardiomyopathy.^23^^,^^24^

Interestingly, women with multiple parity (up to five live births) tolerated pregnancy and the peripartum period well, and complications did not increase with increasing parity. Furthermore, parity did not increase the risk of PAC as no PAC case occurred in second or subsequent pregnancy. This findings is in contrast to observation from PPCM cohorts, in which a subsequent pregnancy has been associated with recurrence of left ventricular dysfunction.^25^ These findings may further support the concept that PAC in TTNtv carriers represents a clinical entity distinct from PPCM.

### Fetal outcomes

In population-based studies from Europe, preterm birth occurs in approximately 5% to 8% of live births and low birth weight in about 6% to 7%. Fetal mortality rates are reported around 3 per 1,000 births.^26^ In comparison, our cohort showed lower rates of prematurity (1%) and low birth weight (3%), whereas fetal or neonatal mortality occurred in 1% of pregnancies. The reported fetal death occurred in a patient who delivered in the 1970s, with incomplete information available. The mother reported an asymptomatic and uncomplicated pregnancy, suggesting an unlikely association between the fetal death and maternal TTNtv genotype. Our results suggest a low incidence of fetal complications in TTNtv positive women, in line with incidences in the general European population.^26^ However, these findings should be interpreted with caution given the small sample size and the retrospective collection of obstetric outcomes.

### Effect of parity on DCM progression in TTNtv-positive women

Pregnancy is associated with important changes in the cardiovascular system, including increases in cardiac output and heart rate and a reduction in systemic vascular resistance.^27^ These changes could theoretically act as stressors in TTNtv-positive women. Although not triggering PAC, these hemodynamic stressors could affect DCM progression in TTNtv-positive population later in life. However, our results showed that at the last follow-up, almost 2 decades after the last live birth, no significant differences in cardiac dimensions and function were observed between parous and nulliparous women. Importantly, the last cardiological follow-up occurred at a median of 18 years after the first pregnancy. This long interval reduces the likelihood that clinically significant cardiomyopathy developed after pregnancy and remained undiagnosed. Multivariable analyses did not demonstrate significant effect of parity on cardiac dimensions and function after adjustment for medical therapy. At last follow-up, parity did not differ significantly between patients who developed left ventricular systolic disfunction and those who did not.

In single variate analyses, there was a trend toward higher NSVT prevalence in those with 2 live births, whereas this association was not apparent after age adjustment. The natural course of TTNtv includes the risk of arrhythmias with increasing age. Prevalence of AF or VT did not differ when comparing parous with nulliparous TTNtv-positive women. No woman required heart transplantation, and only 1 participant died during follow-up at 86 years of age.

In summary, our results suggested that pregnancy—if not complicated by PAC—did not appear to aggravate or accelerate disease progression in TTNtv-positive women. These findings are supported by previous observations showing a less malignant prognosis in female TTNtv-positive individuals.^18^

### Study Limitations

This was an ambispective study with inherent limitations. The single-center design and the lack of systematically collected data on race and ethnicity limit the generalizability of our findings to broader population. However, the likely predominance of European ancestry might be relevant for the interpretation and contextualization of our findings. Our center is a referral center for cardiomyopathies and for pregnant women with heart disease, which could have biased the reported incidence upward. However, most TTNtv-positive women experienced pregnancy years before the TTNtv diagnosis and were managed mostly at local hospital during pregnancy, limiting this potential selection bias. Symptoms during pregnancy were retrospectively self-reported and may therefore be subject to recall bias. In addition, subclinical, mild, and asymptomatic cases of pregnancy-related cardiomyopathy may have gone unrecognized, particularly in previous pregnancies if symptoms were not severe enough to prompt cardiological evaluation. Most obstetric and fetal outcomes, together with data on pregnancy loss, were also collected retrospectively via health questionnaire; therefore, reporting and recall bias cannot be excluded. This may have led to under-reporting of less severe complications and inaccuracies in timing or clinical details; however, major cardiovascular events were systematically verified through electronic medical records and therefore likely to be more robustly captured. Detailed obstetric parameters such as gestational age at delivery, birthweight, and admission to neonatal intensive care unit were not systematically available in our cohort; therefore, the reported fetal outcomes should be interpreted with caution. However, the 3 patients experiencing PAC were followed up at our center, with available electronic medical records for the analysis. Although PAC can occur after miscarriage or stillbirth, we used the number of previous pregnancies resulting in live births as the exposure variable to compare our incidence with previous studies that calculated PPCM incidence per live birth. Pregnancies were analyzed as independent observations; therefore, potential within-subject correlation in women with multiple pregnancies was not accounted for. Given the limited number of events, multivariable models may be subject to overfitting and should be interpreted with caution. The absence of association between parity and long-term cardiac phenotype should be interpreted with caution given the limited statistical power and the potential for survivorship bias.

The participants in our cohort showed a relatively mild cardiac phenotype. This finding reflects the relatively young median age at the last follow-up and is consistent with the age-related penetrance of TTNtv cardiomyopathy.^19^^,^^28^

## Conclusions

The incidence of PAC among TTNtv-positive women was 1.8% per live birth and 3.9% among parous women, which is higher than the 0.02% to 0.1% incidence reported for PPCM in the European population. These results support a predisposing role of TTNtv variants in the pathogenesis of PAC in an otherwise low-risk population. Our results highlight the importance of preconception counseling and follow-up during pregnancy in TTNtv genotype–positive women. Nevertheless, pregnancy and the postpartum period were generally well tolerated, with no increase in maternal or fetal complications, and no signal of increased risk in subsequent pregnancies. Prior pregnancy and multiparity did not appear to affect the long-term prognosis of TTNtv-related cardiomyopathy. Our findings can help guide counseling of TTNtv genotype–positive women with child wish.

Perspectives **COMPETENCY IN PATIENT CARE AND PROCEDURAL SKILLS:** PAC occurred more frequently in TTNtv-positive women than PPCM in general European population. However, most pregnancies and deliveries were uncomplicated despite nearly one third of women reporting cardiac symptoms during pregnancy. These findings highlight the importance of preconception counseling in women carrying TTNtvs.

**TRANSLATIONAL OUTLOOK:** Repeated pregnancies were not associated with adverse long-term cardiac phenotype after 2 decades of follow-up. This observation is reassuring for TTNtv-positive women wishing to become pregnant. Larger prospective studies are needed to identify predictors of pregnancy-associated cardiomyopathy in TTNtv-positive women and to refine risk stratification and pregnancy counseling in this population.

## Funding support and author disclosures

Haugaa, Castrini, Hasselberg, C Bugge, Five and Aabel were supported by grants from the 10.13039/501100005416Research Council of Norway; Precision Health Center for optimized cardiac care (ProCardio) (Grant #309762), and FRIPRO GENE POSITIVE [Grant #288438]. Dr Aabel has received speaker honoraria from Boehringer-Ingelheim, AstraZeneca, and NovoNordisk; has served on an advisory board for Boehringer-Ingelheim and AstraZeneca; and has served on an adjudication committee for Medtronic. Dr Haugaa has received speakers’ honoraria from Bristol Myers Squibb and advisory board honorarium from Agiana Pharmaceuticals. All other authors have reported that they have no relationships relevant to the contents of this paper to disclose.
