# Supplementary material for: Pregnancy-Associated Cardiomyopathy and Long-Term Cardiac Phenotype in Women With Truncating Titin Variants
Source: JACC Adv. 2026 Jun 24;5(10):102857. doi: 10.1016/j.jacadv.2026.102857 (PMC13699709; doi:10.1016/j.jacadv.2026.102857)
Supplement: Supplementary file 1 [file mmc1.pdf]

Pregnancy History Questionnaire Used for Data Collection

Participants were asked to retrospectively report symptoms, pregnancy complications, and medical evaluations occurring during pregnancy and the postpartum period. If more than one pregnancy occurred, participants were asked to specify the pregnancy number (e.g., first, second, third) in the answer.

Participant Information

Name: ____________________________________

Date of birth: _______________________________

Pregnancy History

1. Have you ever been pregnant?
☐ Yes ☐ No

If yes:

Number of pregnancies: _____________________

Age at each pregnancy (years):

Number of deliveries: _____________________

Date(s) of delivery:

---

2. Have you experienced a miscarriage before or after 12 weeks of gestation?
☐ Yes ☐ No

If yes, how many? Please specify:

Delivery and Neonatal Outcomes

3. Did you or your baby experience complications during delivery?
☐ Yes ☐ No

If yes, please indicate the complication(s):
(Please specify the pregnancy number if applicable.)

☐ Low birth weight
☐ Bleeding
☐ Preterm birth (gestational week ______)

☐ Cesarean section (C-section)
☐ Other (please specify):

Genetic Diagnosis

4. Were you diagnosed with a Titin (TTN) gene variant in relation to a pregnancy?
☐ Yes ☐ No

Symptoms During Pregnancy

5. Did you experience any of the following symptoms during pregnancy or within 6 months after delivery?

Symptom	Yes	No
Palpitations	☐	☐
Chest pain	☐	☐
Fainting (syncope)	☐	☐
Shortness of breath (dyspnea)	☐	☐
Near-fainting (presyncope)	☐	☐
Other (please specify)	☐	☐

If other, please describe:

---

6. Did these symptoms occur during all pregnancies or only specific pregnancies?

Cardiac Evaluation

7. Were you diagnosed with heart disease during pregnancy or within the postpartum period?
☐ Yes ☐ No

8. Were you evaluated by a doctor or hospitalized for this condition?
☐ Yes ☐ No

9. Did you undergo an echocardiogram during pregnancy or after delivery?
☐ Yes ☐ No ☐ I do not remember

Medication Use

10. Did you take any medications during pregnancy or the postpartum period?
☐ Yes ☐ No

If yes, please specify:

11. Do you remember the dose(s)?

12. Additional comments:

Supplemental table 1: Pathogenic and likely pathogenic variants found in our cohort of TTNtv-positive women.

Variant (nucleotide)	**Variant (protein)**	**Number of patients**	**Type of variant**	**exon (e)/ TTN band**	**Comments**
c.102523C>T	R34175*	1	TTNtv	358/A-band	stop gain
c.102718_102719del	T34340Hfs*5	1	TTNtv	358/A-band	frameshift
c.102796_102798delinsTATA	N34266Yfs*8	11	TTNtv	358/A-band	frameshift
c.11497_11515del	M3833Cfs*3	1	TTNtv	48/I-band	frameshift
c.13450del	E4484Kfs*2	6	TTNtv	48/I-band	frameshift
c.14020_14027dup	C4677Sfs*7	1	TTNtv	48/I-band	frameshift
c.44284C>T	R14762X	1	TTNtv	240/I-band	stop gain
c.61876C>T	R20626*	2	TTNtv	304/A-band	stop gain
c.3523+1G C	-	1	TTNtv	intron 21/near Z-disk	canonical splice donor
c.50170C>T	R16724*	1	TTNtv	266/A-band	stop gain
c.43359del	P14453Lfs*4	3	TTNtv	235/I-band	frameshift
c.49171C>T	R16391*	1	TTNtv	262/A-band	stop gain
c.51444del	D17149Ifs*4	1	TTNtv	272/A-band	frameshift
c.51532A>T	K17178*	1	TTNtv	272/A-band	stop gain
c.54999dup	p.Cys18334Metfs*5	1	TTNtv	283/A-band	frameshift
c.55577G>A	W18526*	2	TTNtv	287/A-band	stop gain
c.58016del	L9339Cfs*11	1	TTNtv	296/A-band	frameshift
c.59626+1G>A	-	1	TTNtv	intron 301/A-band	canonical splice donor
c.59848C>T	R19950*	6	TTNtv	302/A-band	stop gain
c.59926+1G>A	-	2	TTNtv	intron 302/A-band	canonical splice donor
c.62722C>T	R20908*	1	TTNtv	304/A-band	stop gain
c.64453_64454del	R21485Nfs*2	2	TTNtv	309/A-band	frameshift
c.64826del	T21609Rfs*3	1	TTNtv	310/A-band	frameshift
c.65698dup	D21900Gfs*40	2	TTNtv	313/A-band	frameshift

c.69415C>T	R23139*	1	TTNtv	325/A-band	stop gain
c.68286_68289dup	W22764Nfs*4	1	TTNtv	321/A-band	frameshift
c.72669del	D24224Ifs*8	1	TTNtv	326/A-band	frameshift
c.74338C>T	R24780*	1	TTNtv	326/A-band	stop gain
c.70859_70860dup	P23621Dfs*46	4	TTNtv	326/A-band	frameshift
c.72669del	D24224Ifs*8	2	TTNtv	326/A-band	frameshift
c.72808del	I24270Lfs*11	1	TTNtv	326/A-band	frameshift
c.76156_76157insAG	G25386Efs*31	2	TTNtv	326/A-band	frameshift
c.76085_76086del	L25362Sfs*14	1	TTNtv	326/A-band	frameshift
c.77248C>T	R25750*	1	TTNtv	326/A-band	stop gain
c.78564del	E26189Nfs*17	1	TTNtv	326/A-band	frameshift
c.81572G>A	W27191*	2	TTNtv	326/A-band	stop gain
c.82240C>T	R27414*	2	TTNtv	326/A-band	stop gain
c.83284A>T	R27762*	2	TTNtv	326/A-band	stop gain
c.83407del	V27803Sfs*68	1	TTNtv	326/A-band	frameshift
c.8442del	F2814Lfs*12	1	TTNtv	326/A-band	frameshift
c.87118+1G>T	-	1	TTNtv	intron 327/A-band	canonical splice donor
c.87848del	L29283Yfs*118	1	TTNtv	329/A-band	frameshift
c.89017C>T	R29673*	3	TTNtv	333/A-band	frameshift
c.90696del	R30233Dfs*57	2	TTNtv	335/A-band	frameshift
c.91097_91100del	R30366Ifs*24	2	TTNtv	335/A-band	frameshift
c.91715dupA	N30572Kfs*16	4	TTNtv	337/A-band	frameshift
c.93202G>T	E31068*	1	TTNtv	339/A-band	stop gain
c.93990G>A	W31330*	2	TTNtv	339/A-band	stop gain
c.94103_94107del	I31368Sfs*34	1	TTNtv	339/A-band	frameshift
c.94249del	V31417Sfs*40	2	TTNtv	340/A-band	frameshift
c.94437T>G	Y31479*	3	TTNtv	340/A-band	stop gain
c.95479C>T	Q31827*	1	TTNtv	344/A-band	stop gain
c.95623dup	E31875Gfs*4	4	TTNtv	344/A-band	frameshift

c.96870_96871del	E32290Dfs*56	1	TTNtv	347/A-band	frameshift
c.97129_97130dup	L32379Hfs*4	9	TTNtv	351/A-band	frameshift
c.97847C>A	S32616*	1	TTNtv	351/A-band	stop gain
c.98451del	W32817*	1	TTNtv	352/A-band	stop gain
c.94437T>G	Y31479*	1	TTNtv	340/A-band	stop gain
